# Genomic ascertainment refines the population prevalence and phenotype associations of germline *TERT* variants

**DOI:** 10.1371/journal.pgen.1012352

**Published:** 2026-11-05

**Authors:** Marena R. Niewisch, Sherifa A. Akinniyi, Kelvin C. de Andrade, Jung Kim, Logan P. Zeigler, Jeremy S. Haley, Gretchen Urban, David J. Carey, Douglas R. Stewart, Sharon A. Savage

**Affiliations:** 1 Department of Pediatrics and Adolescent Medicine, University Medical Center Ulm, Ulm, Germany; 2 Division of Cancer Epidemiology and Genetics, National Cancer Institute, National Institutes of Health, Bethesda, Maryland, United States of America; 3 Cancer Genomics Research Laboratory, Leidos Biomedical Research, Frederick National Laboratory for Cancer Research, Frederick, Maryland, United States of America; 4 Department of Genomic Health, Weis Center for Research, Geisinger Medical Center, Danville, Pennsylvania, United States of America; Baylor College of Medicine, UNITED STATES OF AMERICA

## Abstract

**Background:**

Individuals with deleterious germline variants in telomerase (encoded by *TERT*) are at high risk of pulmonary fibrosis (PF), liver disease (LD), bone marrow failure, head/neck squamous cell carcinoma (HNSCC), and other complications. Clinically ascertained cohorts are subject to biased ascertainment of affected individuals and families with early onset, complex disease. This study used genomic ascertainment to determine prevalence of deleterious *TERT* variants and the associated risks of telomere biology disorder (TBD) phenotypes.

**Methods:**

*TERT* variants were extracted from exome sequencing data in UK Biobank (UKB, 469,377 exomes) and Geisinger-Regeneron DiscovEHR collaboration (DiscovEHR, 170,503 exomes). Cases were individuals with heterozygous deleterious *TERT* variants classified per modified ACMG/AMP criteria. Controls excluded rare, exonic, and splice site *TERT* variants. Electronic health records were assessed for TBD-related ICD10 codes. Case-control comparisons were done separately for UKB and DiscovEHR, accounting for smoking, alcohol, BMI, sex, genetic ancestry, and birthyear.

**Results:**

There were 64 deleterious *TERT* variants in 203 UKB participants and 32 in 138 DiscovEHR participants. Prevalence estimates were 1:2,312 in UKB and 1:1,235 in DiscovEHR. Risk of PF was significantly increased for *TERT* heterozygotes compared with controls (UKB: odds ratio [OR] 10.9 95% confidence interval [CI]: 6.11-19.45, p < 0.001; DiscovEHR: OR 6.64, 95% CI: 3.31-13.3, p < 0.001). Deleterious *TERT* variants were also associated with higher risk of LD (OR 2.4, 95% CI:1.25-4.59, p = 0.038) and HNSCC (OR 5.75, 95% CI: 2.36-14.02, p = 0.001) in UKB but not in DiscovEHR.

**Conclusions:**

Rare deleterious *TERT* variants were present in 0.04 to 0.08% of the populations studied. Genomic ascertainment shows a predominance of pulmonary phenotypes, with PF associated with deleterious *TERT* variants in both cohorts. Associations with LD and HNSCC were observed only in UKB, possibly reflecting cohort differences including healthy volunteer bias. These findings illustrate the clinical importance of genetic diagnosis, and need for TBD-specific clinical trials.

## Author summary

Telomeres are protective structures at the ends of chromosomes that shorten with age and are extended by telomerase, encoded by *TERT*. Rare germline variants in *TERT* cause telomere biology disorders, a group of conditions associated with pulmonary fibrosis, liver disease, bone marrow failure, and elevated cancer risk. Because prior research has relied on cohorts referred for genetic testing based on clinical presentation, the true prevalence of deleterious *TERT* variants in the general population is unclear. We analyzed exome sequencing and linked clinical data from two large population-based biobanks (UK Biobank and Geisinger-Regeneron DiscovEHR) to answer this question. Variants were classified as deleterious using standardized criteria from the American College of Medical Genetics and Genomics. Deleterious *TERT* variants were present in approximately 1 in 1,200–1 in 2,300 individuals, a higher prevalence than previously estimated. Individuals with *TERT* variants were more likely to have pulmonary fibrosis than controls. Increased risks of liver disease and head and neck squamous cell carcinoma were also detected, though only in one cohort. These results underscore the utility of population-scale genomic data for characterizing disease risk associated with rare genetic variants.

## Introduction

Telomeres are long (TTAGGG)_n_ nucleotide repeats and a protein complex at chromosome ends essential for genomic stability that shorten with each cell division due to the inability of DNA polymerase to fully replicate DNA ends [1]. Telomerase reverse transcriptase, encoded by *TERT*, extends telomeric nucleotide repeats in germ and stem cells, however *TERT* is repressed in differentiated cells. Germline pathogenic or likely pathogenic variants (P/LP) in *TERT* are associated with short telomeres, increased risk of pulmonary fibrosis (PF), bone marrow failure (BMF), cancer, and other complications [2–13].

*TERT* is one of at least 15 telomere maintenance genes with rare germline P/LP variants associated with the telomere biology disorders (TBDs) [14–16]. Genotype-phenotype studies suggest that heterozygous *TINF2,* X-linked recessive (*DKC1*), or autosomal recessive (e.g. *TERT, CTC1, PARN, RTEL1, WRAP53*) P/LP variants are more likely to be associated with childhood onset TBDs, including dyskeratosis congenita [17]. Heterozygous P/LP variants in *TERT, TERC, RTEL1, PARN*, or *ZCCHC8* are more likely to present in adulthood with isolated BMF, PF, liver disease, and/or cancer, and usually have an autosomal dominant inheritance pattern [18]. Notably, heterozygous P/LP *TERT* variants are present in 10–20% of PF families, making them one of the most common causes of familial PF [10,11,19,20].

The age at onset of *TERT-*related manifestations can be highly variable, even within families, due to incomplete penetrance and variable expressivity [14,21]. To date, studies of TBDs have focused on clinically ascertained individuals and families and consequently are often biased towards severe and/or early-onset disease. The prevalence of TBDs due to P/LP *TERT* variants in the general population is currently unknown [22].

Linking genomic databases with clinical data provides the opportunity to identify individuals with P/LP variants of interest and then objectively assess their associated phenotypes [23]. This genomic ascertainment or “genome-first” approach minimizes ascertainment bias caused by defining an inherited disorder by clinical manifestations and can provide estimates of population penetrance and variant-associated disease risk. This approach has been highly informative in phenotype characterization and quantification in cancer predisposition syndromes [24,25].

This study is the first to determine the population prevalence of rare germline *TERT* variants and explore their TBD-associated phenotypes using data from the United Kingdom Biobank (UKB) and the Geisinger-Regeneron DiscovEHR collaboration (DiscovEHR) and provides key insights as to the public health impact of *TERT*-associated TBDs.

## Methods

### Ethical statement

This research was conducted using the UKB Resource under application #54389. MyCode project is approved under protocol #2006–0258. NCI-Geisinger collaboration is approved under IRB #2021–0818 and contract #75N91021C00006.

### Study participants

UK Biobank is a voluntary, population-based cohort of approximately 500,000 participants aged 40–69 years at recruitment, recruited between 2006 and 2010 from across the UK [26]. This study used whole exome sequencing (WES) variant data from peripheral blood DNA available for research use in total of 469,377 individuals from the UKB linked electronic health records (EHR), cancer and death registries [27–29]. We used the UKB data released on July 12, 2024 [23,30] (UKB application number 54389). Participants withdrawn from the UKB study as of August 2025 were excluded from this analysis.

DiscovEHR consists of 170,503 unselected participants (age 0–90 + years) recruited from in- and outpatient sites within the Geisinger, a large regional medical system headquartered in Danville, Pennsylvania (PA), USA, serving central and northeast PA [31,32]. Informed consent was obtained from DiscovEHR participants or their legal guardians to undergo WES and SNP array genotyping by Regeneron Genetics Center, Tarrytown, NY, USA and to have their health information linked to Geisinger for research purposes (protocol #2006–0258) [32]. De-identified participant data for this study were shared with the National Cancer Institute (NCI) (IRB #2021–0818 and contract #75N91021C00006).

Genetic ancestry for UKB was determined as described previously [33] and grouped as European non-Finnish (UK, Poland, Italy) versus Others (Ashkenazi, Caribbean, China, India, Iran, Nigeria, Others). For DiscovEHR genetic ancestry was determined as previously described and grouped European versus Others (African, East Asian, Admixed American Ancestry, South Asian, Unknown) [32].

### *TERT* variant curation

UKB germline *TERT* variants were obtained from field 23157, population level exome OQFE variants, and pVCF format, final exome (Goldilocks) release [34,35]. DiscovEHR germline *TERT* variants were obtained from WES done as part of the DiscovEHR collaboration as previously described [32]. Variants were annotated with ANNOVAR and snpEFF using transcript NM_198253.3 with genome build GRCh38. Only exonic nonsynonymous, and splice site variants with a minor allele frequency (MAF) <1% were considered (see Fig 1 for additional details). Copy number variants were not evaluated.

**Fig 1:**
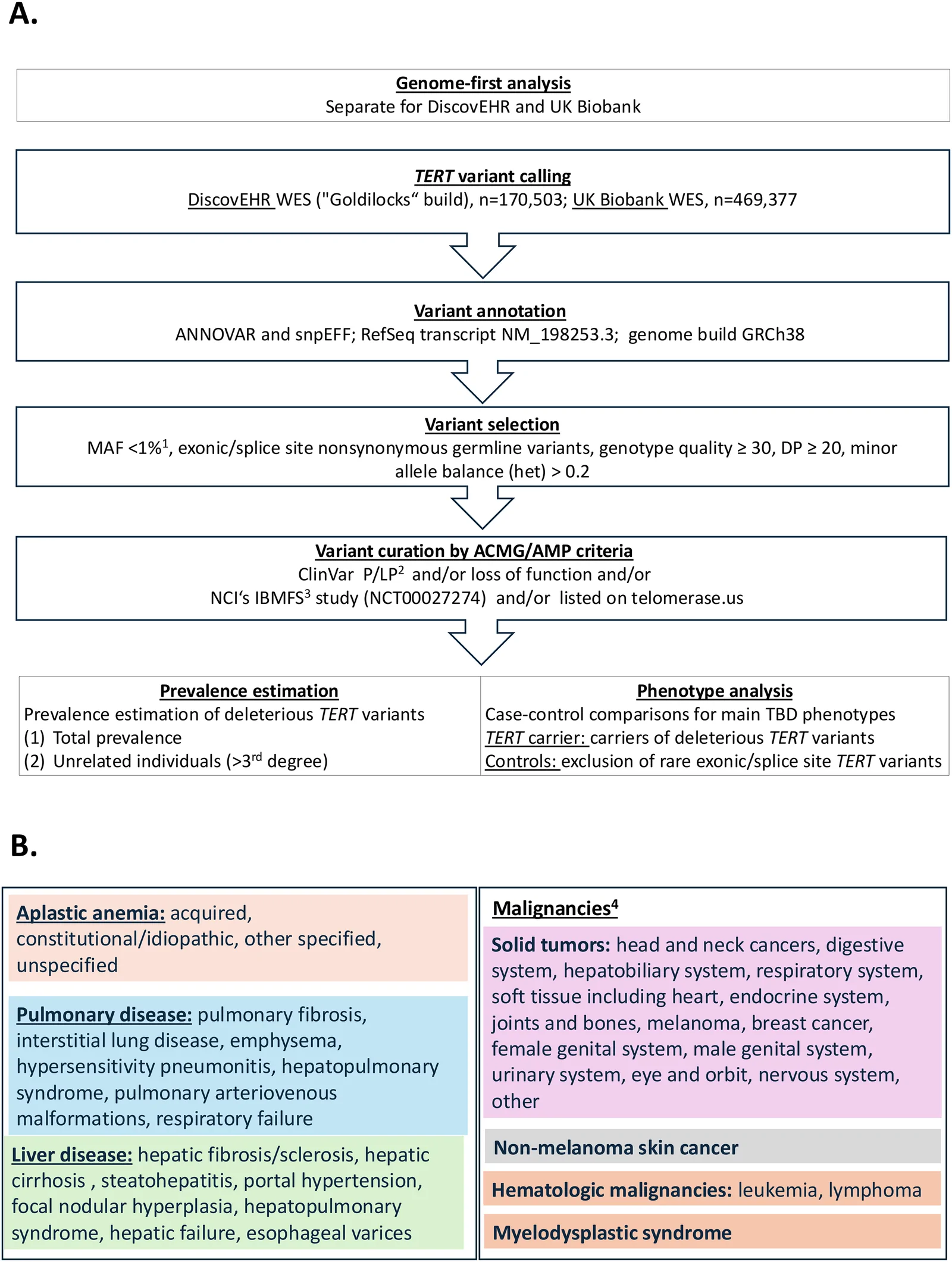
Variant curation, prevalence estimation and phenotype-association analysis of deleterious *TERT* germline variants. **A. Applied genome-first approach.** ^1^MAF, minor allele frequency: < 1% in ExAC_nontcga_ALL, exome AF, exome AF_popmax,exome non_cancer_AF_popmax, controls_AF_popmax, genome AF, genome AF_popmax, genome non_cancer_AF_popmax, genome controls_AF_popmax, and esp6500siv2_all, 1000g2015aug_all. ^2^P/LP, pathogenic/likely pathogenic by at least 1 star rating. ^3^National Cancer Institute’s Inherited Bone Marrow Failure Syndromes Study. **B. Genome based phenotype-analysis.** Schematic depiction of the 4 considered phenotype categories based on known TBD-associated complications. Aplastic anemia ICD10 codes: D60.0, D60.8, D60.9, D61.0, D61.3, D61.8, D61.9. Pulmonary disease: J84.1, J84.0, J84.8, J84.9, J43.0, J43.1, J43.2, J43.8, J43.9, J98.2, J98.3, J67.9, K76.81, Q27.30, J96, Liver disease: K74.0, K74.1, K74.2, K74.3, K74.4, K74.5, K74.6, K75.8, K76.0, K76.6, K76.89, K76.81, K72.0, K72.1, K72.9, I85. Malignancies: C00-C14, C15-C17, C18-C21, C26.0, C22-C25, C26.8, C26.9, C48, C30-C34, C38, C39, C47, C49, C37, C73-C75, C40-C41, C43, C50, C51-C52, C54-C58, C60-C63, C64-C68, C69, C70-C72, C76, C97, C26.1, C45, C46. Non-melanoma skin cancer: C44. Hematologic malignancies: C81-C96, D47. Myelodysplastic syndrome: D46. *Exclusion of benign neoplasms, carcinomas in situ (D00-D36), secondary neoplasms (C77-C80), neoplasms of uncertain behavior (D37-D45, D48, cervix uteri (C53, since largely HPV-associated).

Following the genome-first approach, variants were classified independent of clinical information from UKB and DiscovEHR, and associated phenotypes were assessed only afterwards.

*TERT* variants were classified using modified ACMG/AMP criteria as previously described [21]. Briefly, we included loss of function (LOF) and/or missense variants deemed P/LP in ClinVar (database retrieved March 14, 2023) with at least a one-star rating. We added *TERT* variants present in TBD affected individuals in the NCI Inherited Bone Marrow Failure Syndromes (IBMFS) cohort (**S1 Fig****) [**36] and/or reported in the literature based on a review of the Telomerase Database [37] (last accessed in 09/2025) if they were not in ClinVar. Variants of uncertain significance (VUS) in ClinVar were included if previously reported in TBD cases and had evidence of pathogenicity by ACMG/AMP criteria (VUS-P) (see **Table A in** S1 Appendix for applied ACMG/AMP criteria and Niewisch et al^21^). All P/LP and VUS-P variants were grouped and deemed “deleterious” for further analyses.

### *TERT* variant prevalence estimation

Prevalence estimates were calculated for individuals harboring deleterious *TERT* variants in the DiscovEHR and UKB populations separately. We calculated prevalence in each cohort overall and after removing individuals related up to the third degree by randomly selecting one carrier per family. A third prevalence estimate removing the Ashkenazi Jewish founder c.3150G > C (p.K1050N) variant was also calculated [38].

For UKB, the function “ukb_gen_samples_to_remove() from the R package “*ukbtools*” (version 0.11.3.9) was used to identify individuals for removal and thereby obtain the largest possible set of unrelated participants [39]. For DiscovEHR, to remove related kinship, pairs related up to third degree (minimum PI_HAT = 0.1875) were used to create a graph of all relatives. Custom functions employing the network library in python were used. For each connected component (i.e., family), the node (i.e., patient) with the greatest number of edges (i.e., relatives) was removed. This was repeated until no edges remain in the connected component [40]. Confidence Intervals (Cis) for binomial proportions were generated using the function proportion_confint() from the *statsmodels* Python package (version 0.13.2) [41].

### Phenotype analyses

Phenotype analyses were based on the International Statistical Classification of Diseases and Related Health Problems, Tenth Revision (ICD-10) [42]. We focused on the four main TBD-associated disease categories, malignancies, severe BMF (aplastic anemia), pulmonary, and liver diseases (Fig 1**, Table B in** S1 Appendix), because there is no specific TBD ICD code [21,36,43,44]. Single-lineage cytopenias or macrocytosis as markers of mild or early bone marrow failure, were not assessed, as exclusion of other underlying causes is not feasible at a population-based level. Benign neoplasms, carcinomas *in situ* (D00-D36), secondary neoplasms (C77-C80), neoplasms of uncertain behavior (D37-D45, D48) were excluded for cancer analysis. Due to its largely HPV-associated etiology cervix uteri cancers (C53) were not included [45]. Phenotype queries in UKB included field 40006 (type of cancer: ICD10), 41270 (diagnoses: ICD10), 40001 (underlying [primary] cause of death: ICD10), and 40002 (contributory [secondary] causes of death: ICD10) [26,30]. For DiscovEHR, relevant clinical data were obtained from the Geisinger EHR and/or cancer registry data collected from January 1, 1996, through December 31, 2022 with additional chart review as needed. The Geisinger cancer registry has been maintained since the 1940s and contains malignant tumor diagnoses for all patients diagnosed or treated at Geisinger facilities. Since there is no central death registry within Geisinger, cause of death was determined by chart review.

Age at diagnosis was not analyzed separately, as the available dates (self-reported or first recorded entry in UKB; first admission to the Geisinger health system in DiscovEHR) do not reliably reflect the date of clinical diagnosis.

Telomere length (TL) was measured by qPCR in UKB participant blood-derived DNA and data obtained from field 22192 (Z-adjusted T/S log). TL percentiles for each age were generated based on data from 443,815 controls with available TL measurements, using the age at recruitment (field 21022) as reference for TL of *TERT* heterozygotes [30,46]. TL data are not available in DiscovEHR.

All statistical analysis were conducted separately for the DiscovEHR and UKB populations. Cases were defined as individuals harboring a heterozygous deleterious *TERT* germline variant. We did not identify individuals with biallelic deleterious *TERT* germline variants in either population. Controls in each cohort consisted of individuals without rare (MAF < 1%) coding (exonic or splice-site) *TERT* germline variants. All other rare coding and intronic *TERT* variants were excluded from the control group, regardless of ClinVar pathogenicity classification or variant QC status, since variants currently classified as benign or of uncertain significance may later be reclassified as pathogenic. (UKB controls: 457,261 individuals; DiscovEHR controls:160,680 individuals).

The first occurrence of any relevant ICD-10 diagnosis within every category (Fig 1) per participant was considered to avoid duplicate diagnoses. Association between phenotypes and *TERT* variant status was estimated by calculating odds ratios (OR) and 95% confidence intervals [CIs] determined by logistic regression comparing *TERT* heterozygotes with controls using SAS Enterprise Guide v 8.3 (8.3.0.103) for DiscovEHR and the *statsmodels* Python package (version 0.13.2) for UKB [41]. Multivariable logistic regression models were fitted separately in each cohort and for each disease outcome, with TERT heterozygote status as the primary exposure and disease status as the outcome. Models were adjusted for sex, genetic ancestry, smoking status, and alcohol consumption as categorical covariates, and BMI and year of birth as continuous covariates. Year of birth was included to account for age as well as birth-cohort and temporal trends that may reflect changes in clinical practice and disease ascertainment over time. P-values were adjusted for multiple comparisons using the FDR-BH method [47]. Forest plots depicting ORs were generated using forestploter in RStudio v 4.1.2.

In the UKB, missing BMI values were imputed using the mean BMI within each cohort. For categorical variables (smoking history and alcohol history), missing values were included as a separate “missing” category. Kaplan-Meier survival analyses were used for all-cause mortality estimation with events alive or deceased at last contact. As this is a population-based analysis there was no lost to follow-up status available. For missing demographic data in DiscovEHR, the population mean of the respective population was utilized for continuous variables. Missing data of discrete variables was not included in calculations. The number of missing values is indicated if applicable. Kaplan-Meier survival analyses were used for all-cause mortality estimation with events alive or deceased at last contact. Only events occurring 3 months or after first admission to DiscovEHR facilities are included in survival analyses. Data were truncated to individuals with current age < 85 years. Hazard ratios (HR) were calculated using the Cox Proportional-Hazards (coxph) model with the same covariates as those used for OR calculations (sex, year of birth, genetic ancestry, BMI, and smoking and alcohol use). Log-rank test for equality was applied to compare differences between the curves for *TERT* heterozygotes versus controls. Kaplan-Meier and Cox Proportional-Hazards analyses were conducted using R version 4.1.2. Continuous variables are presented as means and standard deviations (SD) and were compared using Student’s t-test. Year of birth is presented as median and range and was compared using the Mann–Whitney U test. Categorical variables were compared using the chi-squared test. All p-values were two-sided, and p < 0.05 was considered statistically significant.

## Results

### Population prevalence of deleterious germline *TERT* variants

Heterozygous deleterious variants were identified in 341 individuals (203 UKB, 138 DiscovEHR). There were 80 distinct deleterious germline variants distributed across all TERT protein domains without specific cohort-based clustering (71 P/LP, 9 VUS-P; Fig 2A), including 41 LOF, (25 frameshift, 13 stopgain, and three canonical splice variants) and 39 missense variants. Forty-seven variants were previously reported in individuals with TBD-associated phenotypes, and 25 variants were listed in ClinVar as P/LP (Table A in S1 Appendix). Sixteen variants (2 LOF, 14 missense) were present in both the UKB and DiscovEHR (Fig 2A). The most common variants were c.508G > A; p.V170M (n = 44), c.1589C > T; p.P530L (n = 42), c.3150G > C; p.K1050N (n = 34), c.2105C > T; p.P702L (n = 20), and c.2329G > A; p.V777M (n = 10). No individuals with biallelic deleterious variants were identified.

**Fig 2:**
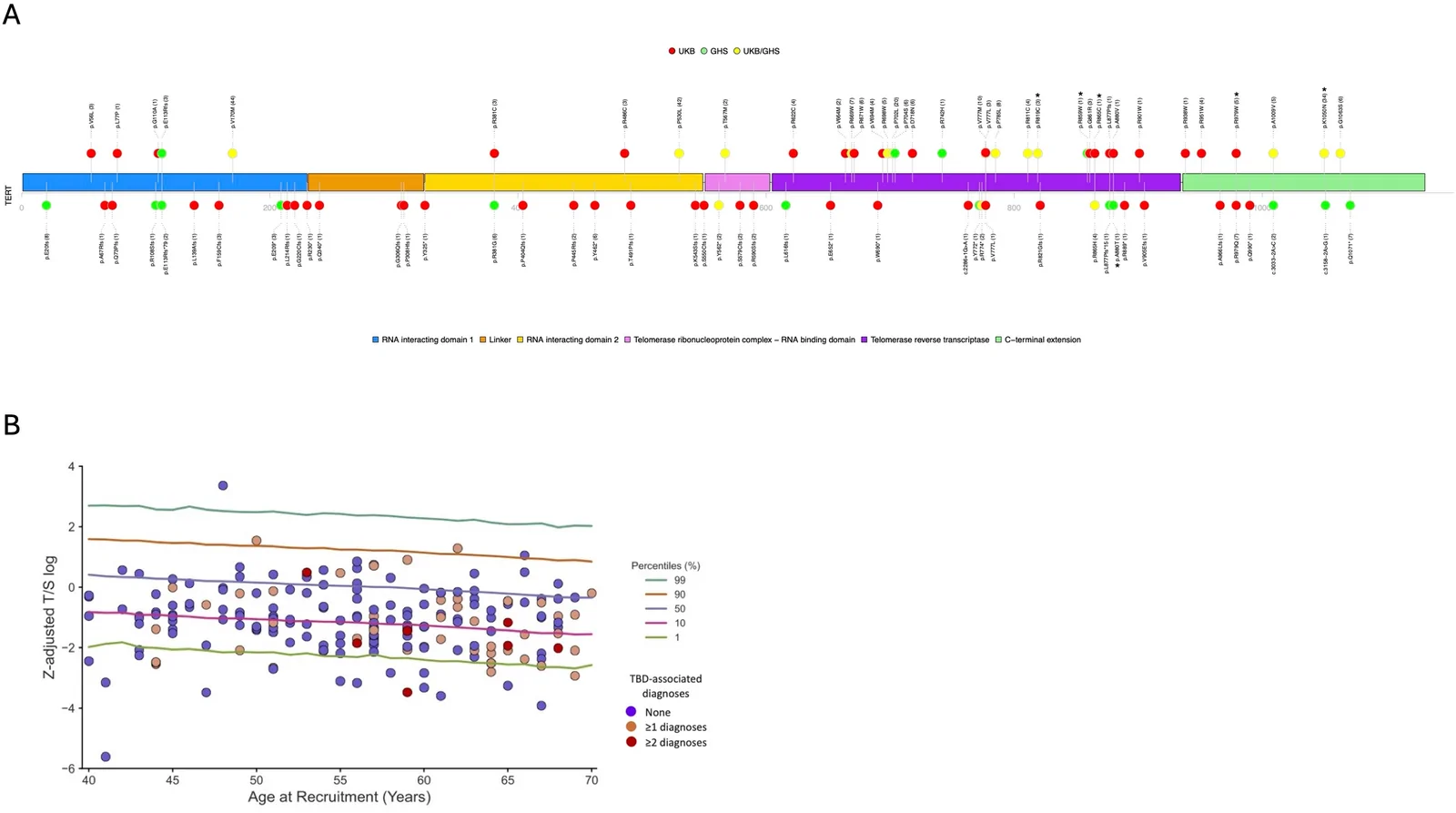
Heterozygous deleterious *TERT* germline variants detected in DiscovEHR and the UK Biobank. **A. Schematic depiction of amino acid changes caused by deleterious germline *TERT* variants across the TERT protein.** For cDNA change see S1 Appendix Table A. Red dots indicate variants identified solely in the UK Biobank *TERT* carrier cohort, green dots highlight variants only identified in the DiscovEHR *TERT* carrier cohort, yellow dots depict variants present in both cohorts. Numbers in parentheses reflect the total number of individuals harboring each variant. *denotes variants also present in clinically diagnosed TBD individuals participating in the National Cancer Institute’s Inherited Bone Marrow Failure Syndromes Study **B. Telomere lengths of *TERT* heterozygotes in UK Biobank.** Telomere length (TL) percentiles for each age were calculated based on Z-adjusted T/S log of qPCR TL (data field p22192) and age at recruitment (data field p21022) available for 443,815 controls. The dots represent TL of 194 *TERT* heterozygotes measured in samples collected at recruitment (n = 9 TL measurement missing). *TERT* variant heterozygotes with pulmonary disease, liver disease, cancer (including skin cancer), or aplastic anemia were highlighted in either orange (at least 1 diagnosis) or red dots (at least 2 different diagnoses). Blue dots represent *TERT* heterozygotes who were not diagnosed with any of these major manifestations.

The overall prevalence of deleterious *TERT* variants was 1:2,312 (95% CI 2,033-2,681) in the UKB and 1:1,235 (95% CI 1,059-1,483) in DiscovEHR (Table 1). After removing related individuals up to the third-degree, prevalence estimates were 1: 2,198 (95% CI 1,929-2,552) in the UKB and 1:1,029 (95% CI 865-1,267) in DiscovEHR. Removal of the Ashkenazi Jewish founder variant c.3150G > C (n = 29 participants in the UKB and n = 5 in Geisinger) resulted in a population prevalence was 1:2,698 (95% CI 2,349-3,168) in UKB and 1:1,282 (95% CI 1,095-1,544) in DiscovEHR. After accounting for relatedness and the founder variant, conservative prevalence estimates were 1:2,542 (95% CI 2,212-2,989) and 1:1,078 (95% CI 904-1,336) (UKB and DiscovEHR, respectively).

**Table 1:** *TERT* variant counts and prevalence in the UK Biobank and DiscovEHR cohorts.

	UK Biobank	DiscovEHR
Total participants	469,377^a^	170,503
**unrelated**^**b**^	437,305	111,079
**<18 years of age**	0	3,453
**Distinct deleterious variants**	64	32
**frameshift**	19	6
**stop gain**	10	5
**canonical splice site**	1	2
**missense**	34	19
**Total heterozygotes** (excluding c.3150G > C) ^c^	203 (174)	138 (133)
**Total unrelated heterozygotes**^**b**^ (excluding c.3150G > C) ^c^	199 (172)	108 (103)
**Prevalence Estimates [95% CI]** ^**c**^		
All	1:2,312 [2,033-2,681]	1:1,235 [1,059-1,483]
All, excluding c.3150G > C ^c^	1:2,698 [2,349-3,168]	1:1,282 [1,095-1,544]
Unrelated	1: 2,198 [1,929-2,552]	1: 1,029 [865-1,267]
Unrelated, excluding c.3150G > C	1:2,542 [2,212-2,989]	1:1,078 [904-1,336]

^a^Number of participants as of August 2025

^b^Unrelated up to the third degree

^c^Ashkenazi Jewish founder variant c.3150G > C: n = 29 carriers within the UK Biobank and n = 5 carriers within DiscovEHR

### Characteristics of the study populations

In the UKB, there were 64 distinct deleterious *TERT* variants in 203 individuals (mean age at recruitment 56.0 ± 7.9 years, 54.2% female). There were 138 individuals harboring 32 distinct deleterious *TERT* variants in DiscovEHR (mean age 40.6 ± 19.2 years, 55.1% female). There was a statistically significant difference between ancestry in UKB cases compared with controls (p < 0.001) which was not significant after removal of Ashkenazi ancestry from the “Other” category (p = 0.904) (Table 2). All *TERT* heterozygotes in DiscovEHR were of European ancestry.

**Table 2:** Characteristics of individuals with heterozygous deleterious *TERT* variants and controls.

	UK Biobank			DiscovEHR		
	*TERT* heterozygotes	Controls^a^	p-value	*TERT* heterozygotes	Controls^a^	p-value
**Number of participants**	203	457,261		138	160,680	
**Age at enrollment, mean (SD)** ^**b**^	56.0 (7.9)	56.5 (8.1)	0.3	40.6 (19.2)	41.6 (18.6)	0.531
**Female, N (%)**	110 (54.2)	247,759 (54.2)	1	76 (55.1)	97322 (60.6)	0.187
**Year of birth, median (range)**	1951 (1938-1969)	1950 (1934-1971)	0.28	1962 (1947-1974)	1961 (1949-1977)	0.811
EUR genetic ancestry, N (%)*	176 (86.7)	428,650 (93.7)	<0.001	138 (100.0)	152041 (94.6)	0.005
**BMI, mean (SD),** [N missing]^**c**^	26.8 (4.5), [0]	27.4 (4.8), [1849]	0.04	30.7 (8.6), [4]	31.0 (7.8), [4722]	0.6938
**Smoking history (ever), N (%),** [N missing]^**c**^	119 (58.6), [0]	272,269 (59.5), [2284]	0.844	84 (61.3), [1]	91736 (57.4), [940]	0.357
**Alcohol history (ever), N (%)**, [N missing]^**c**^	196 (96.6), [0]	436,037 (95.4), [1163]	0.521	90 (65.7), [1]	101411 (63.5), [963]	0.593
**Deceased, N (%)**	27 (13.3)	40104 (8.8)	0.031	25 (18.1)	18016 (11.2)	0.010
**Age at death, mean (SD)**	70.1 (8.3)	71.0 (7.6)	0.54	76.9 (12.9)	73.9 (13.5)	0.282

a For controls from both populations, all heterozygotes of rare (MAF < 1%) exonic or splice site *TERT* variants were excluded, regardless of QC criteria or ClinVar classification

b Age at recruitment for the UK Biobank, minimum encounter age for Geisinger

c As described in the Methods, for UK Biobank missing BMI values were imputed using the mean BMI within each cohort. For categorical variables (smoking history and alcohol history), missing values were included as a separate ‘missing’ category. For missing demographic data in Geisinger, the population mean of the respective population was utilized for continuous variables. Missing data of discrete variables was not included in calculations. The number of missing values is indicated if applicable.

*For UKB included Non-Finish European: United Kingdom, Polish, Italian. All other ancestries were grouped as “Others” and included: Ashkenazi, Caribbean, China, India, Iran, Nigeria. For DiscovEHR genetic ancestry was defined as European or “Others.” Others included: African, East Asian, Admixed American Ancestry, South Asian, Unknown.

Abbreviations: EUR, European; N, number; SD, standard deviation

### Genomically ascertained heterozygotes with deleterious *TERT* variants are at high risk of pulmonary disease

There was a statistically significantly increased risk of pulmonary disease (PD, including PF, see **Table B in** S1 Appendix) in *TERT* variant heterozygotes compared with controls in both cohorts (UKB: OR 3.41 [95% CI: 2.1-5.53], p < 0.001; DiscovEHR: OR 1.95 [95% CI: 1.26-3.02], p = 0.025, Fig 3, **Table C in** S1 Appendix). After excluding respiratory failure, the risks of PD increased in UKB to an OR 5.52 (95% CI: 3.31-9.21, p < 0.001) and in DiscoverEHR to an OR 2.97 (95% CI: 1.83-4.8, p < 0.001). We separately analyzed PF as a discrete phenotype: there was very high risk of PF for heterozygotes compared with controls (UKB: OR 10.9 [95% CI: 6.11-19.45], p < 0.001; DiscovEHR: OR 6.64 [95% CI: 3.31-13.3], p < 0.001). This is also reflected in the higher percentage of PF diagnoses among *TERT* carriers compared to controls (UKB: 6.4% vs 0.7%; DiscovEHR: 6.5% vs 1.0%; **Table C in** S1 Appendix).

**Fig 3:**
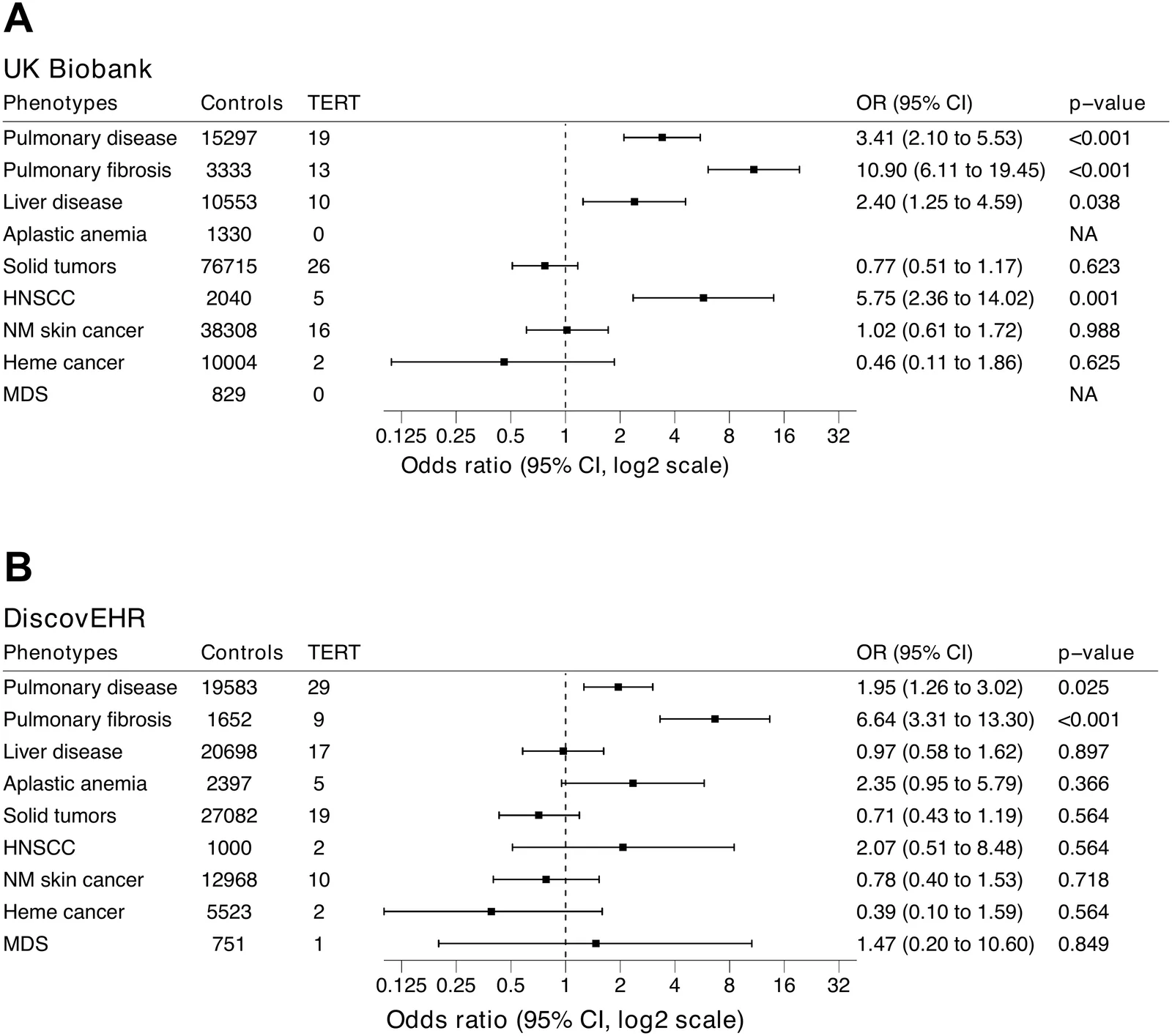
Clinical phenotypes deleterious *TERT* variant heterozygotes. Calculation of odds ratios accounting for sex, year of birth, genetic ancestry, BMI, smoking status, alcohol history. p-value adjusted for multiple comparisons (FDR-BH method). CI, confidence interval; Heme cancer, hematologic cancer; HNSCC, head and neck squamous cell carcinoma; MDS, myelodysplastic syndrome; NM, non-melanoma; OR, odds ratio. **A.** UK Biobank risk of TBD clinical complications in heterozygotes compared with controls. **B.** DiscovEHR risk of TBD clinical complications in heterozygotes compared with controls.

### Deleterious *TERT* variants and Telomere length

qPCR based TL of 194 UKB individuals harboring deleterious *TERT* germline variants were predominantly between the 1^st^ and 50^th^ percentiles for age (Fig 2B). Five of seven individuals with ≥ 2 TBD phenotypes had TL below the 10th percentile for their age. Phenotypes of these five individuals included pulmonary emphysema and bladder cancer (n = 1), PF and skin squamous cell carcinoma (SCC; n = 2), interstitial pulmonary disease and esophageal varices (n = 1), and liver cirrhosis and lung cancer (n = 1). There were 2 individuals with ≥ 2 TBD phenotypes and TL > 10^th^ percentile; one had pulmonary emphysema and bile duct cancer, the other PF and skin SCC. Of note, one *TERT* carrier harboring a frameshift *TERT* variant exhibited TL > 95^th^ percentile (Fig 2B); this carrier had no history of TBD-associated manifestations such as pulmonary, hematologic or hepatic phenotypes (see **Table B in** S1 Appendix for included ICD-10 codes).

### Deleterious *TERT* variants are associated with TBD phenotypes

Given the multisystem nature of TBDs, we next assessed whether *TERT* variant heterozygotes had increased risk of LD, BMF, and/or cancer compared with controls. UKB participants with deleterious *TERT* variants had an increased risk of LD (OR 2.4 [95% CI:1.25-4.59], p = 0.038), but this finding was not present in DiscovEHR (Fig 3, **Table C in** S1 Appendix). There was no increased risk of aplastic anemia/BMF or other cytopenias in *TERT* variant heterozygotes compared with controls in either cohort. The risks of solid tumors, hematologic malignancies, MDS, and non-melanoma skin cancers were also not increased in either cohort. Analyses of cancer subgroups revealed an increased risk of head/neck squamous cell carcinoma (HNSCC) in UKB *TERT* heterozygotes (OR 5.75 [95% CI: 2.36-14.02], p = 0.001) but not in DiscovEHR. However, DiscovEHR chart review revealed that one of the two *TERT* heterozygotes with HNSCC was diagnosed at the unusually young age of 26 years (**Table D in** S1 Appendix).

Overall, 58 of 203 (28.6%) UKB and 56 of 138 (40.6%) DiscovEHR heterozygotes were diagnosed with at least one of the four primary TBD-associated phenotypes (PD, LD, BMF, and/or cancer). Seven UKB individuals (3.4%) and 20 (14.5%) DiscovEHR individuals had two or more ICD10 diagnoses in these categories. We conducted exploratory analyses of other TBD-associated manifestations including gastrointestinal telangiectasias, pulmonary arteriovenous malformations, avascular necrosis, immunodeficiency, and bone fractures, but did not identify statistically significant associations (**Table C in** S1 Appendix).

### Pulmonary disease is a common cause of death in individuals with heterozygous deleterious *TERT* variants

We observed decreased survival of UKB heterozygotes compared with controls (HR 1.77 [95% CI 1.21-2.58], log-rank p = 0.003; Fig 4), but the age at death was not significantly lower in heterozygotes than controls (70.1 ± 8.3 vs 71.0 ± 7.6 yrs, p = 0.54). Of note, in 27 deceased *TERT* heterozygotes, 12 had PD as a cause of death, including eight with PF (**Table E in** S1 Appendix). The DiscovEHR population had the same tendency toward increased mortality for heterozygotes compared with controls, but these observations were not statistically significant (HR 1.5 [95% CI 0.9-2.3], log-rank p = 0.097). There was no significant difference in age at death for 25 deceased DiscovEHR *TERT* heterozygotes versus controls (76.9 ± 12.9 vs 73.9 ± 13.5 years, p = 0.282). Details on death causes were based on chart review in DiscovEHR and were notable for a predominance of PD related death causes (28%), followed by cancer (16%). In 13 of 25 individuals cause of death was unknown but a history of severe PD was reported in six of the 13, including one individual receiving lung transplant for PF 15 years prior to death.

**Fig 4:**
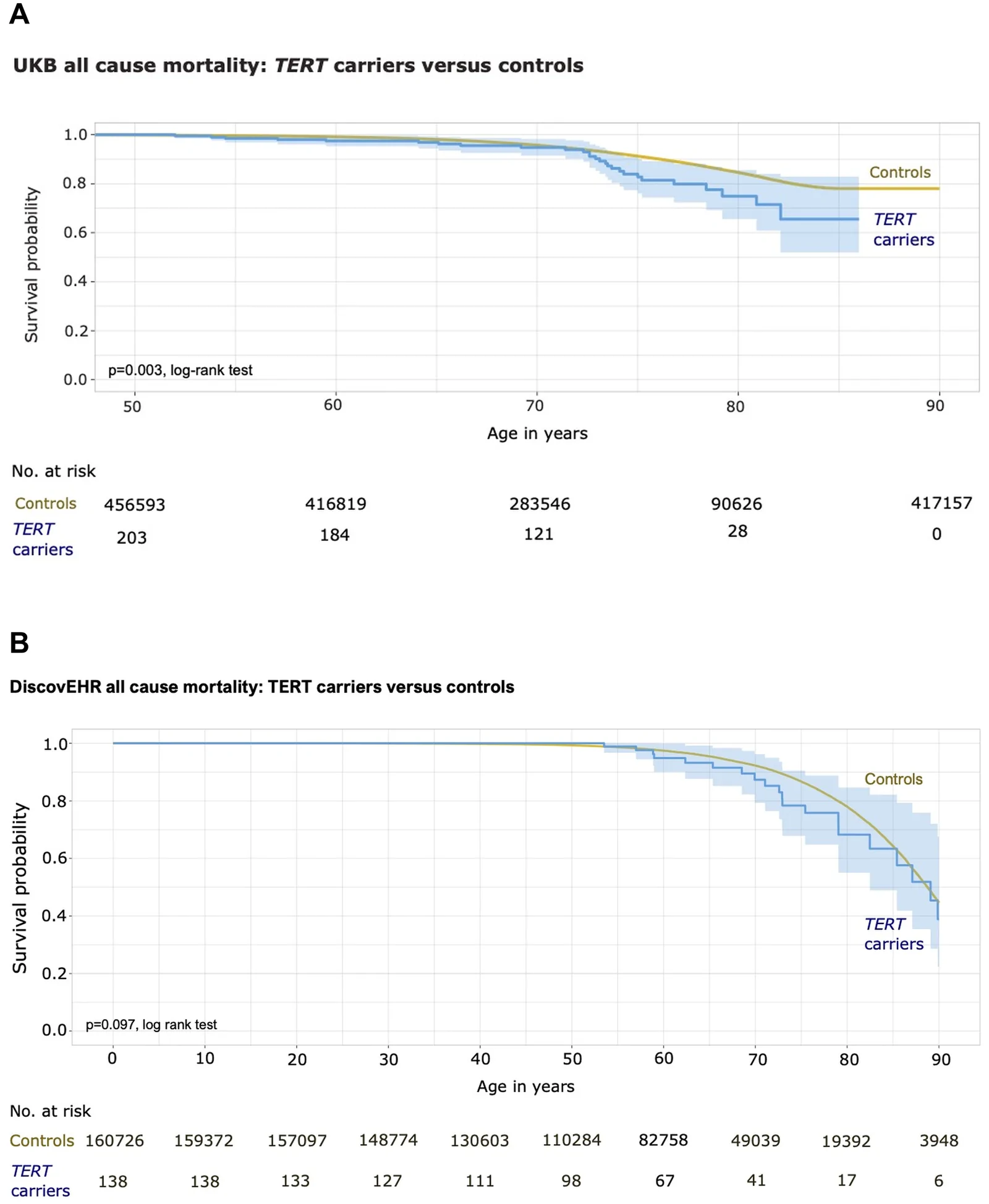
All-cause mortality and death causes in deleterious *TERT* variant heterozygotes compared with controls. p-values and hazard ratio adjusted for genetic ancestry, sex, year of birth, BMI, smoking, alcohol history. Controls excluded heterozygotes of any rare (minor allele frequency <1%) germline *TERT* variant. **A.** UK Biobank. **B.** DiscovEHR.

## Discussion

Individuals with TBDs due to heterozygous *TERT* variants usually present as adults with fewer clinical manifestations than those with classic dyskeratosis congenita [18,21]. Most studies of *TERT-*related phenotypes have relied upon clinical ascertainment of individuals with genetic diagnosis and assessment of family members, a practical approach but one subject to ascertainment of individuals and families with early onset, complex disease [2,4,21,48]. Using two distinct genomically-ascertained adult cohorts, this study provides the first population-based prevalence estimates of deleterious *TERT* variants and their associated clinical consequences.

We were somewhat surprised to find the prevalence of unrelated individuals with heterozygous deleterious *TERT* variants to range from 1 in 1,078–2,542 in the Geisinger and UKB cohorts, respectively, after excluding the founder Ashkenazi Jewish c.3150G > C variant [38]. To date, overall TBD prevalence estimate of 1 in 1,000,000 has been based on expert opinion and included consideration of the complex, multi-system nature of TBDs. Similar to other genomic ascertainment studies [24,25,40], this difference may be due, at least in part, to lack of genetic testing in individuals with mild or only one TBD manifestation, limited cascade testing of family members, and/or limited assessment in other cohorts.

To test our hypothesis that genomically ascertained deleterious *TERT* variant heterozygotes were more likely to have TBD manifestations compared with controls, we focused the main phenotype on analyses on the four major adult-onset TBD complications (PD, LD, BMF, and cancer). The risk of PD overall and specifically PF was markedly higher in *TERT* heterozygotes than controls (2–3-fold and 6–10-fold great, respectively). The higher OR for PF compared with PD overall indicates that the association between *TERT* variants and PD is driven largely by PF, although non-fibrotic manifestations may contribute. Prior family-based studies estimate that 10–20% of familial PF is associated with *TERT* variants [49–51]. Lung transplantation in patients with PF due to *TERT* variants is frequently complicated by prolonged cytopenias due to the risk of bone marrow failure underlying the TBDs [52–54]. Our data confirm the role of *TERT* variation in PF etiology and provide the first population-level data to further justify genetic testing in individuals and families with PF, which can guide informed decision making and optimize patient care. Genetic diagnosis is especially important as there are very limited data on whether individuals with TBD-related PF respond to antifibrotic therapy [55]. The data shown here support the implementation of a TBD-specific clinical trial.

TBD patient cohorts including *TERT* heterozygotes are often comprised of individuals with early-onset, severe disease and studied by groups with specific expertise (e.g., BMF). Notably, this study did not identify associations between *TERT* heterozygotes and hematopoietic manifestations such as severe BMF, leukemia, or MDS. Clinical follow-up in this population-based study relied on diagnoses captured by EHR, which may lead to underestimation of MDS and/or other illnesses. Data from clinically ascertained cohorts, show that severe BMF is a primary manifestation of early-onset TBDs. In contrast, individuals with heterozygous TERT genotypes tend to manifest later in life [21]. Somatic *TERTp* and *PPM1D* mutations have been associated with poorer survival in TBDs, suggesting a compensatory response to critical telomere shortening rather than uniform protection [56]. In UKB WGS data analysis, somatic TERTp mutations have been identified as a novel age-associated driver of clonal hematopoiesis [57], and telomere-maintenance-related clonal hematopoiesis was enriched among individuals with shorter genetically predicted telomere length [58]. Thus, somatic events may play a role in expressivity and penetrance of hematologic phenotypes of underlying *TERT* germline variants.

Idiopathic liver fibrosis/cirrhosis has been reported as a first manifestation of adult-onset TBD, including that associated with *TERT* variants [17]. *TERT* heterozygotes in the UKB had an increased risk of LD compared with controls, but this finding was not present in DiscovEHR.

We did not observe a statistically significant increased risk of malignancy in these genomically ascertained cohorts. As we have previously shown, the highest cancer risk in TBD patients may be for individuals with autosomal recessive or X-linked disease [59]. Therefore, genomically ascertained *TERT* heterozygotes may not have a significantly increased cancer rate compared with the general population. It is important to note that risk of HNSCC was increased in *TERT* heterozygotes in the UKB and there was one early onset HNSCC (26 years of age) in a DiscovEHR heterozygote. Prior studies of clinically ascertained TBDs due to heterozygous P/LP variants, including but not limited to *TERT*, found a 2.56-fold increased risk of cancer compared with the general population; in that study, six of 22 *TERT* variant carriers had a history of cancer (none with HNSCC) [36]. Similarly increased risk of HNSCC was also reported in a study of 226 individuals with TBDs (including 99 *TERT* carriers) [60]. These data suggest that consideration of a TBD is warranted in young individuals (i.e., < 40 years) with HNSCC and that cancer screening in individuals with heterozygous deleterious *TERT* variants should be considered.

This study also provided an opportunity to assess whether *TERT* heterozygotes had distinct causes of death or poorer overall survival than non-heterozygotes. The causes of death in both cohorts were remarkable for a tendency towards pulmonary causes, suggesting the link between PD and poor outcomes in *TERT* heterozygotes. Overall survival was also poorer in heterozygotes compared with control but the age at death was not significantly different.

The strengths of this study include genomic ascertainment by carefully and conservatively curated variants, and detailed phenotypic data analysis. We acknowledge that this study was limited by the older age of individuals in both cohorts and thus may be biased towards adult-onset TBD manifestations. Clinically ascertained data show that heterozygous *TERT* carriers typically present later in life. Our approach might specifically have under ascertained individuals with biallelic *TERT* variants, who generally manifest disease in childhood and are therefore less likely to be captured in population-based adult cohorts [21]. It is likely that the genome-first approach used here may underrepresent phenotype penetrance because of the healthy volunteer effect notable in the UKB and the older age at assessment of individuals in UKB and DiscoverEHR [61]. The ICD10 code-based phenotype analysis approach may miss subclinical disease or lack specificity. Differences in geography, including differences in environmental exposures, healthcare systems, diagnostic coding practices, and recruitment/ascertainment strategy could contribute to cross-cohort differences such as the elevated LD and HNSCC risk noted in UKB but not in DiscovEHR data.

The UKB TL data provided an opportunity to assess TL in *TERT* heterozygotes compared with age-matched controls. The UKB TL data was generated by qPCR, which has limited sensitivity in diagnosing TBDs [62–64]. Our data did not find a clear association between TL and *TERT* variant status but assay limitations and variability in *TERT*-associated TBDs preclude further assessment [62–64].

Genomic ascertainment found the prevalence of deleterious *TERT* variants in two population cohorts ranging between 1 in 1,078 and one in 2,542. Pulmonary phenotypes, including PF, predominate in individuals with heterozygous *TERT* P/LP variants illustrating the importance of genetic diagnosis and need for TBD-specific clinical trials. Large, longitudinal studies are necessary to further our understanding of the phenotype manifestations of different TBD genes – which is essential for a personalized medicine approach for this patient population.

## Supporting information

S1 Appendix **Table A:** Deleterious germline *TERT* variants detected in UK Biobank (UKB) and DiscovEHR participants. **Table B:** ICD-10 codes used for focused phenotype analysis based on known telomere biology disorder-associated manifestations. **Table C:** Phenotypes reported in individuals with heterozygous deleterious germline *TERT* variants compared with controls. **Table D:** Mean ages at cancer diagnosis in *TERT* carriers versus controls in the DiscovEHR population. **Table E:** Classification of causes of death in 27 deceased *TERT* carriers within the UK Biobank. Each row represents one deceased individual.
(DOCX)

S1 Fig Locations of pathogenic/likely pathogenic *TERT* germline variants detected in UK Biobank or DiscovEHR that were previously reported in clinical TBD cases in the literature. (TIFF)
