# Supplementary material for: Genomic ascertainment refines the population prevalence and phenotype associations of germline TERT variants
Source: PLoS Genet. 2026 Nov 5;22(11):e1012352. doi: 10.1371/journal.pgen.1012352 (PMC13739231; doi:10.1371/journal.pgen.1012352)
Supplement: Supplementary file 1 [file pgen.1012352.s001.docx]

**S1 Appendix**

**Genomic ascertainment refines the population prevalence and phenotype associations of germline *TERT* variants**

Marena R Niewisch ^1,2&*^, Sherifa A Akinniyi ^2&^, Kelvin C de Andrade^2^, Jung Kim^2^, Logan P Zeigler ^3^, Jeremy S Haley^4^, Gretchen Urban ^4^, David J Carey^4^, Douglas R Stewart^2^, Sharon A Savage^2*^

& contributed equally; * corresponding authors

^1^ Department of Pediatrics and Adolescent Medicine, University Medical Center Ulm, Ulm, Germany

^2^ Division of Cancer Epidemiology and Genetics, National Cancer Institute, National Institutes of Health, Bethesda, Maryland, United States of America

^3^ Cancer Genomics Research Laboratory, Leidos Biomedical Research, Frederick National Laboratory for Cancer Research, Frederick, Maryland, United States of America

^4^ Department of Genomic Health, Weis Center for Research, Geisinger Medical Center, Danville, Pennsylvania, United States of America

| **Table A:** Deleterious germline *TERT* variants detected in UK Biobank and DiscovEHR participants | | | | | | | | | | |
| --- | --- | --- | --- | --- | --- | --- | --- | --- | --- | --- |
| Variant characteristics | | | N carriers | | | Presence in cohorts, databases or literature | | | ACMG/AMP | |
| **cDNA change** | **AA change** | **Functional classification** | **UKB** | **GHS** | **NCI** | **Telomerase Database** | **Literature PMID** | **ClinVar** | **Applied codes** | **Classification** |
| c.1333delinsAGACC | p.P445Rfs*95 | Frameshift | 2 | 0 | N | N | - | NA | PVS1, PM2 | LP |
| c.1386_1387delinsG | p.Y462* | Stopgain | 6 | 0 | N | N | - | NA | PVS1, PM2 | LP |
| c.1471_1472delinsC | p.T491Pfs*18 | Frameshift | 1 | 0 | N | N | - | P/2943992 | PVS1, PS1_Supporting, PM2 | LP |
| c.1628_1629delinsG | p.K543Sfs*6 | Frameshift | 1 | 0 | N | N | - | NA | PVS1, PM2 | LP |
| c.1648_1650delinsT | p.S550Cfs*78 | Frameshift | 1 | 0 | N | N | - | NA | PVS1, PM2 | LP |
| c.1211delinsAACCACGCGCAGTGCCC | p.P404Qfs*140 | Frameshift | 1 | 0 | N | N | - | LP/3385331 | PVS1, PM2 | LP |
| c.1735_1737delinsT | p.S579Cfs*49 | Frameshift | 2 | 0 | N | N | - | NA | PVS1, PM2 | LP |
| c.1770_1774delinsC | p.R590Sfs*3 | Frameshift | 2 | 0 | N | N | - | NA | PVS1, PM2 | LP |
| c.1837_1841delAGGCC | p.L616Dfs*11 | Frameshift | 0 | 1 | N | N | - | P/2944431 | PVS1, PM2, PS1_Supporting | P |
| c.199delinsCG | p.A67Rfs*125 | Frameshift | 1 | 0 | N | N | 27836952 | P/1070657 | PVS1, PS1_Supporting, PM2 | P |
| c.218delinsCCCCGCCGCCCCCTCCTTCCGCCA | p.Q73Pfs*13 | Frameshift | 1 | 0 | N | N | - | NA | PVS1, PM2 | LP |
| c.C1141T | p.R381C | Missense | 3 | 0 | N | Y | 26024875 | VUS/3907281 | PS4_Supporting, PM2, PP2, PP3 | VUS-P |
| c.C1141G | p.R381G | Missense | 0 | 6 | N | Y | 26024875 | VUS/937135 | PS4_Supporting, PM2, PP2, PP3 | VUS-P |
| c.2461_2463delinsG | p.R821Gfs*48 | Frameshift | 1 | 0 | N | N | - | P/1457059 | PVS1, PM2, PS1_Moderate | P |
| c.2630_2635delinsC | p.L877Pfs*15 | Frameshift | 1 | 0 | N | N | - | P/2008972 | PVS1, PS1_Supporting, PM2 | P |
| c.2714_2716delinsA | p.V905Efs*133 | Frameshift | 1 | 0 | N | N | - | NA | PVS1, PM2 | LP |
| c.2896_2897delinsC | p.A966Lfs*15 | Frameshift | 1 | 0 | N | N | - | NA | PVS1, PM2 | LP |
| c.C1456T | p.R486C | Missense | 3 | 0 | N | Y | 17460043, 27540018, 20502709, 28192371 | LP,VUS/39101 | PVS1, PM2, PS1_Supporting | LP |
| c.C1589T | p.P530L | Missense | 3 | 39 | N | Y | 26024875, 23335200, 22664374, 21520173 | VUS/242218 | PS3_Supporting, PS4_Moderate, PM2, PP2, PP3 | LP |
| c.321_322insT | p.R108Sfs*84 | Frameshift | 0 | 1 | N | N | - | NA | PVS1, PM2 | LP |
| c.336delC | p.E113Rfs*15 | Frameshift | 0 | 3 | N | Y | 17392301 | VUS/4056823 | PVS1, PS4_Supporting, PM2 | LP |
| c.415delinsGC | p.L139Afs*53 | Frameshift | 1 | 0 | N | N | - | NA | PVS1, PM2 | LP |
| c.474_476delinsT | p.F159Cfs*32 | Frameshift | 3 | 0 | N | N | 30995915 | NA | PVS1, PS4_Supporting, PM2 | LP |
| c.57_58delCG | p.E20Gfs*171 | Frameshift | 0 | 8 | N | N | - | P/1994215 | PVS1, PS1_Supporting, PM2 | P |
| c.641_642delinsG | p.L214Rfs*137 | Frameshift | 1 | 0 | N | N | - | NA | PVS1, PM2 | LP |
| c.658_660delinsT | p.G220Cfs*25 | Frameshift | 1 | 0 | N | N | - | NA | PVS1, PM2 | LP |
| c.916delinsCAG | p.G306Qfs*46 | Frameshift | 1 | 0 | N | N | - | NA | PVS1, PM2 | LP |
| c.923_924delinsA | p.P308Hfs*43 | Frameshift | 1 | 0 | N | N | - | P/2928164 | PVS1, PM2, PS1_Moderate | P |
| c.C1018T | p.Q340* | Stopgain | 1 | 0 | N | N | - | NA | PVS1, PM2 | LP |
| c.C1700T | p.T567M | Missense | 1 | 1 | N | Y | 23538340 | LP/265267 | PS1_Moderate, PM2, PS3_Supporting, PS4_Supporting | LP |
| c.C1864T | p.R622C | Missense | 4 | 0 | N | Y | 25612863, 27836952, 33214205 | VUS,LP/659164 | PS4_Supporting, PM1_Supporting, PM2, PP3, PP2 | VUS-P |
| c.C2005T | p.R669W | Missense | 4 | 3 | N | Y | 32315675, 36028256, 32214205, 29281671, 28192371, 40311650 | LP,VUS/539196 | PM2, PS4_Supporting, PM1_Supporting, PP2, PP3 | VUS-P |
| c.C2011T | p.R671W | Missense | 6 | 0 | N | Y | 20502709, 27540018, 28192371, 29281671, 34890115, 34890115, 30995915, 37934624 | LP/410693 | PS1_Moderate, PS3_Supporting, PS4_Moderate, PM1_Supporting, PM2, PP2, PP3 | LP |
| c.C2092T | p.R698W | Missense | 3 | 2 | N | Y | 22664374 | VUS/2443278 | PS4_Supporting, PM1_Supporting, PM2, PP2, PP3 | VUS-P |
| c.C2105T | p.P702L | Missense | 1 | 19 | N | Y | 27540018, 20502709, 18635888, 27836952, 28192371 | LP,VUS/ 653732 | PS4_Supporting, PM1_Supporting, PM2, PP2, PP3, PS1_Supporting | LP |
| c.C2110T | p.P704S | missense | 0 | 6 | Y | Y | 30523342, 18042801, 18635888 , 18931339, 27418648, 28099038, 20502709, 28192371, 35776903, 40311650 | P,LP/39108 | PS1_Moderate, PS3_Moderate, PS4_Moderate, PM1, PM2, PP2, PP3, PS3, PP4 | LP |
| c.C2354T | p.P785L | Missense | 5 | 3 | N | Y | 19760749 | VUS/846805 | PM2, PS4_Supporting, PM1_supporting, PP2, PP3 | VUS-P |
| c.C2431T | p.R811C | Missense | 3 | 1 | N | Y | 36028256, 28192371, 17785587, 33214205 | VUS,LP/29900 | PS1_Supporting, PS3_Supporting, PM2, PS4_Moderate, PM1_Supporting, PP2, PP3 | LP |
| c.C2455T | p.R819C | Missense | 1 | 2 | Y | N | 27159321, 39661387, 34852175 | LP,VUS/ 265268 | PS1_Supporting, PS4_Supporting, PM2, PM1_Moderate, PP2_Moderate, PP4_Moderate | LP |
| c.C2316G | p.Y772* | Stopgain | 0 | 1 | N | N | - | NA | PVS1, PM2 | LP |
| c.C3026T | p.A1009V | Missense | 2 | 3 | N | Y | 27836952‚ 30523342, 37934624 | LP/1065578 | PS1_Moderate, PM2, PS4_Supporting, PP3, PP2 | LP |
| c.C2575T | p.R859W | Missense | 0 | 1 | Y | N | 34852175, 40311650 | VUS/229310 | PM1_Moderate, PM2, PP2_Supportive, PP4_Supportive, BP4 | LP |
| c.C2593T | p.R865C | Missense | 1 | 0 | Y | Y | 20502709, 22364217, 28192371 | LP,VUS/986922 | PS1_Moderate, PM2, PM5, PP2, PP3, PP4 | LP |
| c.C2639T | p.A880V | Missense | 1 | 0 | N | Y | 25612863‚ 27836952 | LP,VUS/872809 | PS1_Supporting, PS4_Supporting, PM2, PP2, PP3, PM5 | LP |
| c.C2701T | p.R901W | Missense | 1 | 0 | N | Y | 17785587‚   30603600, 29281671, 28192371 | VUS,LP/29901 | PS1_Moderate, PM2, PS4_Supporting, PS3_Supporting | LP |
| c.C2812T | p.R938W | Missense | 1 | 0 | N | Y | 28099038, 33035329 | LP/916674 | PS1_Moderate, PM2, PS4_Supporting, PP2, PP3 | LP |
| c.C2851T | p.R951W | Missense | 4 | 0 | N | Y | 27540018, 20502709, 17460043, 27836952, 28192371 | VUS,LP/836202 | PS1_Moderate, PS3_Supporting, PS4_Moderate, PM2, PP2 | LP |
| c.C2665T | p.R889* | Stopgain | 1 | 0 | N | Y | 19674077, 37934624 | LP/3065428 | PVS1, PS1_Supporting, PS4_Supporting, PM2, PP2 | LP |
| c.C2935T | p.R979W | Missense | 5 | 0 | Y | Y | 21602826, 15885610, 23901009, 34852175, 33214205, 37934624, 37934624 | LP/39118 | PS3_Supporting, PS4_Moderate | LP |
| c.G2329A | p.V777M | Missense | 9 | 1 | N | Y | 26024875, 30426156 | VUS,LP/436985 | PS1_Supporting, PS3_Supporting, PS4_Supporting, PM2, PP2, PP3 | LP |
| c.G166C | p.V56L | Missense | 3 | 0 | N | Y | 21931702 | NA | PS3_Supporting, PS4_Supporting, PP2, PP3, PM2 | LP |
| c.G1990A | p.V664M | Missense | 2 | 0 | Y | N | 34852175, 33718801 , 37934624 | VUS/431836 | PM1, PM2, PM3, PM5_Supporting, PP2, PP4 | LP |
| c.C2968T | p.Q990* | Stopgain | 1 | 0 | N | Y | 27836952, 33214205, 37934624 | NA | PVS1, PS4_Supporting, PM2_Moderat, PP2 | P |
| c.G2080A | p.V694M | Missense | 4 | 0 | N | Y | 15814878, 23901009, 20502709, 18635888, 18635888, 31943309, 36028256, 28192371, 37934624 | LP,VUS/12731 | PS1_Moderate, PS4_Moderate, PM2, PP2, | LP |
| c.C3211T | p.Q1071* | Stopgain | 0 | 7 | N | N | - | P/937831 | PVS1, PS1_Supporting, PM2 | P |
| c.C688T | p.R230* | Stopgain | 1 | 0 | N | N | - | P/645232 | PVS1, PS1_Supporting, PM2, PP2 | P |
| c.C975A | p.Y325* | Stopgain | 1 | 0 | N | N | - | NA | PVS1, PM2, PP2 | LP |
| c.G2152A | p.D718N | Missense | 6 | 0 | N | Y | 21931702, 30523342, 33709208 | VUS/1433750 | PS3_Supporting, PS4_Supporting, PM2, PM1, PP2, PP3 | LP |
| c.G1954T | p.E652* | Stopgain | 1 | 0 | N | N | - | NA | PVS1, PM2, PP2 | LP |
| c.G2225A | p.R742H | Missense | 0 | 1 | N | Y | 30995915, 33718801, 28099038, 33214205, 37934624, 37934624 | VUS/165378 | PS4_Moderate, PM1_Supporting, PM2, PP2, PP3 | LP |
| c.G2070A | p.W690* | Stopgain | 1 | 0 | N | N | - | NA | PVS1, PM2, PP2 | LP |
| c.G2594A | p.R865H | missense | 3 | 1 | N | Y | 17460043, 22364217, 30523342, 20022961, 23901009, 27540018, 20502709, 33214205, 20502709, 28192371 | P,LP/12736 | PS1_Moderate, PS4_Strong, PM2, PP2, PP3 | P |
| c.G2329C | p.V777L | Missense | 1 | 0 | N | Y | 26024875, 25346280 | VUS/2679141 | PM2, PM5_Supporting, PP2, PS4_Supporting | VUS-P |
| c.G2329T | p.V777L | Missense | 3 | 0 | N | Y | 26024875, 25346280 | VUS/2679141 | PM2, PM5_Supporting, PP2, PS4_Supporting | VUS-P |
| c.G2581A | p.G861R | Missense | 3 | 0 | N | Y | 21349926‚ 21931702, 33709208, 36028256, 28192371 | VUS/471871 | PS4_Supporting, PM1_Supporting, PM2, PP2, PS3_Supporting | LP |
| c.G3150C | p.K1050N | Missense | 29 | 5 | Y | Y | 26024875, 34852175, 33214205, 28192371 | VUS/350518 | PS4_Moderate, PM3_Supporting, PP1, PP2, PP3, PP4 | LP |
| c.G2638A | p.A880T | Missense | 0 | 1 | Y | Y | 23335200, 39661387, 34852175, 39938003, 27836952 | P,LP/ 950401 | PS3_Supporting, PS4_Moderate, PM1_Supporting, PM2, PP2, PP3, PP4 | LP |
| c.G2936A | p.R979Q | Missense | 7 | 0 | N | Y | 26024875 | VUS,LP/506255 | PS1_Supporting, PM2, PS4_Supporting, PP2, BP4_Supporting | VUS-P |
| c.G3187A | p.G1063S | Missense | 2 | 4 | N | Y | 27540018, 20502709, 21931702, 28192371 | LP/658115 | PS1_Moderate, PS3_Supporting, PS4_Supporting, PM2, PP2, PP3 | LP |
| c.G508A | p.V170M | Missense | 37 | 7 | N | Y | 21436073, 24833766, 25741868, 30523342 | VUS/36947 | PS4_Moderate, PM2, PP3, PP2 | LP |
| c.G329C | p.G110A | Missense | 1 | 0 | N | Y | 26024875 | VUS,LP/1331613 | PS1_Moderate, PM2, PP2, PS4_Supporting | LP |
| c.C2320T | p.R774* | Stopgain | 1 | 1 | N | N | 29483670, 37934624 | P/446373 | PVS1, PS1_Moderate, PS4_Supporting, PM2 | P |
| c.T230C | p.L77P | Missense | 1 | 0 | N | N | 33035329 | VUS,LP/916671 | PS1_Moderate, PS4_Supporting, PM2, PP2, PP3 | LP |
| c.2286+1G>A | c.2286+1G>A | splice site | 1 | 0 | N | N | 30995915 | LP/1502782 | PVS1_Strong, PS1_Moderate, PS4_Supporting, PM2, PP2 | LP |
| c.3033-2A>C | c.3033-2A>C | splice site | 0 | 2 | N | N | - | LP/3377095 | PVS1, PS1_Supporting, PM2 | LP |
| c.G625T | p.E209* | Stopgain | 0 | 3 | N | N | - | NA | PVS1, PM2 | LP |
| c.T1686G | p.Y562* | Stopgain | 1 | 1 | N | N | - | NA | PVS1, PM2 | LP |
| c.3158-2A>G | c.3158-2A>G | splice site | 0 | 1 | N | N | - | NA | PVS1, PM2 | LP |
| c.2630_2634delTCACC | p.L877Pfs*15 | Frameshift | 0 | 1 | N | N | - | P/2008972 | PVS1, PS1_Supporting, PM2. | P |
| c.336dupC | p.E113Rfs*79 | Frameshift | 0 | 2 | N | Y | 33214205, 40311650 | P,LP/410651 | PVS1, PS1_Moderate, PS4_Supporting, PM2 | P |

Abbreviations: AA, amino acid; ACMG, American College of Medical Genetics and Genomics; AMP, Association for Molecular Pathology; GHS, Geisinger Health System; ID, identifier; LP, likely pathogenic; N, no; NA, not applicable; NCI, National Cancer Institute; N, number; P, pathogenic; PMID, PubMed Identifier; Telomerase Database, (<https://telomerase.us/>); TBD, telomere biology disorder; UKB, United Kingdom Biobank; VUS, variant of uncertain significance; Y, yes.

**Table B:** ICD-10 codes used for focused phenotype analysis based on known telomere biology disorder-associated manifestations

| **TBD-associated phenotype** | **Included clinical manifestations** | **Queried ICD-10 codes** |
| --- | --- | --- |
| **Pulmonary disease** | Pulmonary arteriovenous malformations, hypersensitivity pneumonitis, interstitial lung disease, pulmonary fibrosis, emphysema, hepatopulmonary syndrome | K76.81, Q27.30, J67.9, J84.1, J84.0, J84.8, J84.9, J43.0, J43.1, J43.2, J43.8, J43.9, J98.2, J98.3 |
| **Aplastic anemia/bone marrow failure** | Chronic acquired pure red cell aplasia; other acquired pure red cell aplasias; acquired pure red cell aplasia, unspecified; constitutional aplastic anemia; idiopathic aplastic anemia; other specified aplastic anemia; aplastic anemia, unspecified | D60.0, D60.8, D60.9, D61.0, D61.3, D61.8, D61.9 |
| **Liver disease** | Hepatic fibrosis, hepatic sclerosis, liver cirrhosis, nonalcoholic steatohepatitis, nonalcoholic fatty liver disease, portal hypertension, focal nodular hyperplasia of liver, hepatopulmonary syndrome, hepatic failure, esophageal varices | K72.0, K72.1, K72.9, K74.0, K74.1, K74.2, K74.6, K74.3, K74.4, K74.5, K75.8, K76.0 , K76.6, K76.89, K76.81, I85 |
| **Malignancies** |  |  |
| Non-hematologic malignancies | Solid tumors regardless of organ system, excluding cervical cancer (C53) and non-melanoma skin cancer | C00-C26, C30-C34, C37-C41, C43, C45-52, C54-C58, C60-C80, C97 |
| Hematologic malignancies | Leukemia, lymphoma | C81-C96; D47 |
| Myelodysplastic syndrome |  | D46 |
| Non-melanoma skin cancer |  | C44 |

**Table C:** Phenotypes reported in individuals with heterozygous deleterious germline *TERT* variants compared with controls.

| **Phenotype subgroups** | **ICD-10 codes** | **DiscovEHR** | | | | **UK Biobank** | | | |
| --- | --- | --- | --- | --- | --- | --- | --- | --- | --- |
|  |  | **N events (%)** | | **adjusted odds ratio (95% CI)** | **p-value** | **N events (%)** | | **adjusted odds ratio (95% CI)** | **p-value** |
|  |  | **Controls,**  **N=160,680** | ***TERT* carriers, N=138** |  |  | **Controls,**  **N=457,261** | ***TERT* carriers, N=203** |  |  |
| **Pulmonary Disease (PD)** | K76.81, Q27.30, J67.9, J84.1, J84.0, J84.8, J84.9, J43.0, J43.1, J43.2, J43.8, J43.9, J98.2, J98.3, J96 | 19583 (12.2) | 29 (21.01) | 1.95 (1.26-3.02) | 0.025 | 15297 (3.3) | 19 (9.4) | 3.41 (2.1-5.53) | <0.001 |
| PD excluding respiratory failure | K76.81, Q27.30, J67.9, J84.1, J84.0, J84.8, J84.9, J43.0, J43.1, J43.2, J43.8, J43.9, J98.2, J98.3 | 10275 (6.394) | 23 (16.66) | 2.97 (1.83-4.8) | <0.001 | 8683 (1.9) | 17 (8.4) | 5.52 (3.31-9.21) | <0.001 |
| Pulmonary fibrosis | J84.1 | 1652 (1.03) | 9 (6.5) | 6.64 (3.31-13.3) | <0.001 | 3333 (0.7) | 13 (6.4) | 10.9 (6.11-19.45) | <0.001 |
| **Liver disease** | K72.0, K72.1, K72.9, K74.0, K74.1, K74.2, K74.6, K74.3, K74.4, K74.5, K75.8, K76.0, K76.6, K76.8 , K76.89, I85 | 20698 (12.8) | 17 (12.3) | 0.97 (0.58-1.62) | 0.897 | 10553 (2.3) | 10 (4.9) | 2.4 (1.25-4.59) | 0.038 |
| **Cytopenia** | D60.0, D60.8, D60.9, D61.0, D61.3, D61.8, D61.9, D64.8. D53.1, D64.9, D69.3, D69.4, D69.6, D70, D72.819 | 52031 (32.4) | 42 (30.4) | 0.904 (0.62-1.32) | 0.779 | 36690 (8.0) | 15 (7.4) | 0.94 (0.56-1.6) | 0.988 |
| Aplastic anemia | D60.0, D60.8, D60.9, D61.0, D61.3, D61.8, D61.9 | 2397 (1.5) | 5 (3.6) | 2.35 (0.95-5.79) | 0.366 | 1330 (0.3) | 0 | NA | NA |
| **Solid tumors** | C00-C26, C30-C34, C37-C41, C43, C45-C52, C54-C58, C60-C80, C97 | 27082 (16.9) | 19 (13.8) | 0.71 (0.43-1.19) | 0.564 | 76715 (16.8) | 26 (12.8) | 0.77 (0.51-1.17) | 0.623 |
| Head and neck cancer | C00-C14 | 1000 (0.6) | 2 (1.5) | 2.07 (0.51-8.48) | 0.564 | 2040 (0.5) | 5 (2.5) | 5.75 (2.36-14.02) | 0.001 |
| Digestive system (esophagus, stomach, small intestine) | C15-17 | 842 (0.5) | 1 | 1.2 (0.2-8.8) | 0.564 | 3256 (0.7) | 0 | NA | NA |
| Colon excluding Rectum | C18, C26.0 | 2239 (1.4) | 2 (1.5) | 0.97 (0.2-4.0) | 0.428 | 7375 (1.6) | 3 (1.5) | 0.99 (0.32-3.11) | 0.988 |
| Rectum and Rectosigmoid Junction | C19-C21 | 1021 (0.6) | 0 | NA | NA | 4748 (1.0) | 0 | NA | NA |
| Cancer of liver and intrahepatic bile duct | C22-C25, C26.8, C26.9, C48 | 1456 (0.9) | 2 | 1.46 (0.4 -6.0) | 0.564 | 4009 (0.9) | 3 (1.5) | 1.83 (0.58-5.76) | 0.625 |
| Malignant neoplasm: Spleen | C26.1 | 3 (0) | 0 | NA | NA | 9 (0.002) | 0 | NA | NA |
| Malignancies of the respiratory system | C30-C34, C38.1-C.38.8, C.39 | 3181 (2) | 4 (2.9) | 1.27 (0.5-3.5) | 0.887 | 6929 (1.5) | 2 (1) | 0.71 (0.18-2.89) | 0.976 |
| Soft tissue including heart | C38.0, C47, C49 | 504 (0.3) | 1 (0.7) | 2.24 (0.3-16.1) | 0.779 | 823 (0.2) | 0 | NA | NA |
| Bones and joints | C40-C41 | 306 (0.2) | 0 | NA | NA | 293 (0.06) | 0 | NA | NA |
| Melanoma | C43 | 2333 (1.5) | 2 | 0.91 (0.2-3.7) | 0.564 | 5868 (1.2) | 0 | NA | NA |
| Mesothelioma | C45 | 36 (0.02) | 0 | NA | NA | 550 (0.1) | 0 | NA | NA |
| Kaposi Sarcoma | C46 | 19 (0.01) | 0 | NA | NA | 21 (<0.1) | 0 | NA | NA |
| Breast cancer | C50 | 5585 (3.5) | 4 (2.9) | 0.74 (0.3-2.1) | 0.887 | 19121 (4.2) | 9 (4.4) | 1.1 (0.57-2.22) | 0.988 |
| Female genital system | C51-52, C54-C58 | 2754 (1.7) | 2 (1.5) | 0.83 (0.2-3.5) | 0.744 | 5279 (1.2) | 0 | NA | NA |
| Male genital system | C60-C63 | 4979 (3.1) | 2 (1.5) | 0.43 (0.1-1.8) | 0.564 | 17266 (3.8) | 1 (0.5) | 0.12 (0.02-0.85) | 0.129 |
| Urinary System | C64-C68 | 3004 (1.9) | 0 | NA | NA | 7216 (1.6) | 2 (1) | 0.67 (0.17-2.72) | 0.949 |
| Eye and Orbit | C69 | 136 (0.1) | 0 | NA | NA | 315 (0.1) | 1 (0.5) | 7.73 (1.08-55.35) | 0.137 |
| Brain and other Nervous System | C70-C72 | 1391 (0.9) | 0 | NA | NA | 1337 (0.3) | 0 | NA | NA |
| Endocrine system | C73-C75, C37 | 2082 (1.3) | 0 | NA | NA | 1190 (0.3) | 0 | NA | NA |
| Other: Malignant neoplasm of other and ill-defined sites , Malignant neoplasms of independent (primary) multiple sites | C76, C97 | 728 (0.5) | 0 | NA | NA | 2010 (0.4) | 1 (0.5) | 1.22 (0.17-8.7) | 0.988 |
| Non-melanoma skin cancer | C44 | 12968 | 10 | 0.78 (0.4-1.53) | 0.564 | 38308 (8.4) | 16 (7.9) | 1.02 (0.61-1.72) | 0.988 |
| **Hematologic malignancies** | C81-C96, D47 | 5523 (3.4) | 2 (1.5) | 0.39 (0.1-1.59) | 0.564 | 10004 (2.2) | 2 (1) | 0.46 (0.11-1.86) | 0.625 |
| **Myelodysplastic Syndrome** | D46 | 751(0.5) | 1 (0.7) | 1.47 (0.2-10.6) | 0.849 | 829 (0.2) | 0 | NA | NA |
| **Vascular disease** | K55.0, K55.1, K55.2. K55.3, K55.8, K55.9. K76.81, K92.2, Q27.30, [H35.0 in update] | 11325 (7.1) | 8 (5.8) | 0.77 (0.37-1.58) | 0.718 | 18517 (4.1) | 5 (2.5) | 0.61 (0.25-1.49) | 0.625 |
| **Avascular Osteonecrosis** | M87.9 | 1313 (1.8) | 1 (0.7) | 0.99 (0.23-22.7) | 0.989 | 735 (0.2) | 0 | NA | NA |
| **Fractures** | S02, S12, S22, S32, S42, S52, S62, S72, S82, S92 | 40088 (24.94) | 44 (31.88) | 1.38 (0.96-1.98) | 0.407 | 42155 (9.2) | 18 (8.9) | 1.01 (0.62-1.64) | 0.988 |
| **Immunodeficiency** | D80, D81, D83, D84, D89.9 | 3510 (2.184) | 2  (1.45) | 0.64  (0.16 – 2.6) | 0.744 | 644  (0.14) | 0 | NA | NA |

Abbreviations: N, number; NA, not applicable/available

**Table D:** Mean ages at cancer diagnosis in *TERT* carriers versus controls in the DiscovEHR population

| **Cancer category** | **ICD 10 group** | **N controls** | **N *TERT* heterozygotes** | **Controls, mean age at cancer diagnosis (SD)** | ***TERT* heterozygotes, mean age at cancer diagnosis (SD)** | **p-value** |
| --- | --- | --- | --- | --- | --- | --- |
| All solid tumors | C00-C26, C30-C34, C37-C41, C43, C45-C52, C54-C58, C60-C80, C97 | 27082 | 19 | 61.34 (13.75) | 64.65(15.02) | 0.294 |
| Head and neck cancer | C00-C14 | 1000 | 2 | 62.2 (13.0) | 52.3 (37.7) | 0.7747 |
| Digestive system (esophagus, stomach, small intestine) | C15-17 | 842 | 1 | 65.9 (12.8) | 52.0 (.) | 0.2769 |
| colon excluding rectum | C18, C26.0 | 2239 | 2 | 65.2 (12.6) | 79.7 (10.3) | 0.1032 |
| Liver and intrahepatic bile duct | C22-C25, C26.8, C26.9, C48 | 1456 | 2 | 66.1 (12.4) | 76.4 (2.3) | 0.2404 |
| Respiratory system | C30-C34, C38.1-C.38.8, C.39 | 3181 | 4 | 67.0 (11.4) | 68.1 (4.5) | 0.8498 |
| Soft tissue including heart | C38.0, C47, C49 | 504 | 1 | 60.5 (16.7) | 52.2 (.) | 0.6177 |
| Melanoma | C43 | 2333 | 2 | 61.4 (14.7) | 48.2 (1.9) | 0.2068 |
| Non-melanoma skin cancers (BCC and SCC) | C44 | 12968 | 10 | 68.2 (12.4) | 63.8 (10.4) | 0.2606 |
| Breast cancer | C50 | 5585 | 4 | 60.5 (12.3) | 59.4 (10.3) | 0.8561 |
| Female genital system | C51-52, C54-C58 | 2754 | 2 | 59.3 (13.0) | 65.0 (15.6) | 0.5386 |
| Male genital system | C60-C63 | 4979 | 2 | 65.7 (10.7) | 74.2 (2.6) | 0.2616 |
| Non-melanoma skin tumors | C44 | 12968 | 10 | 68.21(12.35) | 63.81(10.43) | 0.261 |
| Hematologic malignancies | C81-C96, D47 | 5523 | 2 | 62.85(15.98) | 73.82(7.62) | 0.332 |
| Myelodysplastic Syndrome | D46 | 751 | 1 | 69.72(13.6) | 72.19 (.) | 0.856 |

Abbreviations: BCC, basal cell carcinoma; N, number; SCC, squamous cell carcinoma

**Table E:** Classification of causes of death in 28 deceased *TERT* carriers in the UK Biobank. Each row represents one deceased individual.

| **Primary cause of death (field p40001_i0)** | **Additional listed contributing to death (field p40002_i0_a1)** | **Death category** |
| --- | --- | --- |
| I24.9 Acute ischaemic heart disease, unspecified | R57.0 Cardiogenic shock | Cardiovascular |
| I21.9 Acute myocardial infarction, unspecified | I25.1 Atherosclerotic heart disease | Cardiovascular |
| I25.1 Atherosclerotic heart disease | I25.9 Chronic ischaemic heart disease, unspecified | Cardiovascular |
| I60.9 Subarachnoid haemorrhage, unspecified | I10 Essential (primary) hypertension | Cerebrovascular |
| I61.3 Intracerebral haemorrhage in brain stem |  | Cerebrovascular |
| F01.9 Vascular dementia, unspecified |  | Cerebrovascular |
| C44.1 Skin of eyelid, including canthus | N18.5 Chronic kidney disease, stage 5 | Malignancy |
| C50.9 Breast, unspecified | A41.9 Septicaemia, unspecified | Malignancy |
| C22.1 Intrahepatic bile duct carcinoma |  | Malignancy |
| C22.1 Intrahepatic bile duct carcinoma |  | Malignancy |
| C80.0 Malignant neoplasm, primary site unknown, so stated |  | Malignancy |
| U07.1 COVID-19 virus identified | E11.9 Without complications | Other |
| K70.9 Alcoholic liver disease, unspecified |  | Other |
| K57.8 Diverticular disease of intestine, part unspecified, with perforation and abscess | K57.9 Diverticular disease of intestine, part unspecified, without perforation or abscess | Other |
| L03.9 Cellulitis, unspecified | N17.9 Acute renal failure, unspecified | Other |
| M06.9 Rheumatoid arthritis, unspecified | J84.1 Other interstitial pulmonary diseases with fibrosis | Other, PF contributing |
| J44.1 Chronic obstructive pulmonary disease with acute exacerbation, unspecified | J44.9 Chronic obstructive pulmonary disease, unspecified | Pulmonary |
| J18.9 Pneumonia, unspecified | K92.2 Gastro-intestinal haemorrhage, unspecified | Pulmonary |
| J67.9 Hypersensitivity pneumonitis due to unspecified organic dust | R68.8 Other specified general symptoms and signs | Pulmonary |
| J44.0 Chronic obstructive pulmonary disease with acute lower respiratory infection | J18.0 Bronchopneumonia, unspecified | Pulmonary |
| J84.1 Other interstitial pulmonary diseases with fibrosis | J18.9 Pneumonia, unspecified | Pulmonary, PF |
| J84.1 Other interstitial pulmonary diseases with fibrosis |  | Pulmonary, PF |
| J84.1 Other interstitial pulmonary diseases with fibrosis |  | Pulmonary, PF |
| J84.1 Other interstitial pulmonary diseases with fibrosis |  | Pulmonary, PF |
| J84.1 Other interstitial pulmonary diseases with fibrosis |  | Pulmonary, PF |
| J84.1 Other interstitial pulmonary diseases with fibrosis | M81.9 Osteoporosis, unspecified | Pulmonary, PF |
| J84.1 Other interstitial pulmonary diseases with fibrosis | J44.9 Chronic obstructive pulmonary disease, unspecified | Pulmonary, PF |
| J84.1 Other interstitial pulmonary diseases with fibrosis | I64 Stroke, not specified as haemorrhage or infarction | Pulmonary, PF |
